# Note on the Vitellary Nature of the Corpus Luteum

**Published:** 1847-02

**Authors:** C. D. Meigs

**Affiliations:** Professor of Midwifery in Jefferson Medical College, &c.


2. *Note on the Vitellary nature of the Corpus Luteum.* By C. D. Meigs, M.D., Professor of Midwifery in Jefferson Medical College, &c. (In a letter to the editor.)

Dear Sir:—I made, at the last meeting of the American Philosophical Society, a statement with regard to the Corpus Luteum, to the following effect:

Upon turning the screw of the compressor, while a bit of fresh corpus luteum lies between the plates, I can, with a magnifying power of 500, perceive the escape from the crushed mass of vast numbers of corpuscles resembling, in every particular, the granules and corpuscles of the yelk of an egg.

I have made the observation a great many times—and I

have placed upon the platine, portions both of fresh yelk and fresh corpus luteum of the cow. Upon the most rigorous comparison of the microscopic appearances in each case, I cannot discover any difference, except that from the crushed bit of corpus luteum there escapes with the yelk grains, a certain quantity of detached cellular, or epithelial structure and blood discs.

When a portion of bright yelk, or a portion of equally bright corpus luteum is under the objective, the whole of the transmitted light, before the focus is obtained, is yellow, and alike in both cases, while the transmitted light is also yellow in passing through a single, or three or four separate corpuscles of either substance.

The micrographers have not noticed this appearance. Dr. Bischoff, in his *Entwickelungsgeschicte*, takes notice of numerous punctiform bodies, but does not hint at an idea of their being yelk grains.

The discovery of the vitellary nature of the corpus luteum, if I have really made the discovery, ought to prove interesting to the physiologist and the medicologist. I beg you, therefore, to do me the favor to mention the circumstance in the forthcoming number of your valuable Journal, in order that the microscopic observers may test its truth or error.—*Amer. Jour. of the Med. Sciences.*

